# Supplementary material for: Feasibility of the “Preventing functional decline in acutely hospitalized older patients (PREV_FUNC)” study—A three-armed randomized controlled pilot trial
Source: PLoS One. 2024 Jun 21;19(6):e0304570. doi: 10.1371/journal.pone.0304570 (PMC11192352; doi:10.1371/journal.pone.0304570)
Supplement: S1 File — (PDF) [file pone.0304570.s002.pdf]

admincontrol

2020-06505 0209.pdf

Signers:

Name	*Method*	*Date*
HANNA WERTH	BANKID	2021-02-22 19:17 GMT+1

This document package contains:

- Front page (this page)
- The original document(s)
- The electronic signatures. These are not visible in the document, but are electronically integrated.

This file is sealed with a digital signature. The seal is a guarantee for the authenticity of the document.

Document ID:
D5752B8288274881BE8C65B60255173B

THE SIGNED DOCUMENT FOLLOWS ON THE NEXT PAGE >

Dnr 2020-06505
Lund avdelning 1 medicin

BESLUT
2021-02-09

Sökande forskningshuvudman
Region Stockholm

Forskare som genomför projektet
Anna-Karin Welmer

Projekttitel
Effekt av träning under akut sjukhusvistelse på funktionell förmåga för patienter 75 år och äldre–en pilotstudie

---

Etikprövningsmyndigheten beslutar enligt nedan.

BESLUT

Etikprövningsmyndigheten godkänner den forskning som anges i ansökan.

På Etikprövningsmyndighetens vägnar

Hanna Werth
Ordförande

Beslutet har fattats av följande personer:

Ordförande
Hanna Werth, rådman

Ledamöter med vetenskaplig kompetens
Eva Brun (onkologi)
EwaCarin Ekberg (odontologi)
Maria Haak (hälsovetenskap, gerontologi)
Lars Hagander (pediatrik)
Göran Holst (geriatrisk omvårdnad, vetenskaplig sekreterare)
Ulf Jakobsson (allmänmedicin, psykiatrisk epidemiologi och migration)
Kristina Källén (neurologi)
Maria Landqvist Waldö (psykiatri)
Oonagh Shannon (infektionsmedicin, immunologi, hematologi)
Carl Johan Tiderius (ortopedi, föredragande)

This file is sealed with a digital signature.
The seal is a guarantee for the authenticity of the document.
Document ID:
D5752B8288274881BE8C65B60255173B

Dnr 2020-06505
Lund avdelning 1 medicin

Ledamöter som företräder allmänna intressen
Jerry Bergström
Agnetha Järvegren
Lars Karlsson
Ewa Pihl Krabbe

Beslutet sänds till
Ansvarig forskare: Anna-Karin Welmer
Forskningshuvudmannens företrädare: Martin Annetorp

registrator@etikprovning.se | 010-475 08 00 | Box 2110, 750 02 Uppsala | etikprovning.se

This file is sealed with a digital signature.
The seal is a guarantee for the authenticity of the document.
Document ID:
D5752B8288274881BE8C65B60255173B
