# Supplementary material for: Feasibility of the “Preventing functional decline in acutely hospitalized older patients (PREV_FUNC)” study—A three-armed randomized controlled pilot trial
Source: PLoS One. 2024 Jun 21;19(6):e0304570. doi: 10.1371/journal.pone.0304570 (PMC11192352; doi:10.1371/journal.pone.0304570)
Supplement: S2 File — (DOCX) [file pone.0304570.s003.docx]

Dnr 2020-06505

Lund department 1 medicine

**DECISION**

2021-02-09

**Research Principal**

Stockholm Region

**Researcher carrying out the project**

Anna-Karin Welmer

**Project title**

Effect of exercise during acute hospital stay on functional ability for patients 75 years and older - a pilot study

The Ethics Review Authority decides as below.

**DECISION**

The Ethics Review Authority approves the research specified in the application.

On behalf of the Ethics Review Authority

Hanna Werth

President

The decision has been made by the following people:

**President**

Hanna Werth, councilor

**Members with scientific competence**

Eva Brun (oncology)

EwaCarin Ekberg (odontology)

Maria Haak (health science, gerontology)

Lars Hagander (pediatrics)

Göran Holst (geriatric nursing, scientific secretary)

Ulf Jakobsson (general medicine, psychiatric epidemiology and migration)

Kristina Källén (neurology)

Maria Landqvist Waldö (psychiatry)

Oonagh Shannon (Infectious Diseases, Immunology, Hematology)

Carl Johan Tiderius (orthopedics, rapporteur)

**Members representing the public interest**

Jerry Bergström

Agnetha Järvegren

Lars Karlsson

Ewa Pihl Krabbe

**The decision is sent to**

Responsible researcher: Anna-Karin Welmer

Representative of the Research Principal: Martin Annetorp
