# Supplementary material for: Feasibility of the “Preventing functional decline in acutely hospitalized older patients (PREV_FUNC)” study—A three-armed randomized controlled pilot trial
Source: PLoS One. 2024 Jun 21;19(6):e0304570. doi: 10.1371/journal.pone.0304570 (PMC11192352; doi:10.1371/journal.pone.0304570)
Supplement: S3 File — (PDF) [file pone.0304570.s004.pdf]

BESLUT

2022-01-13

Sökande forskningshuvudman
Region Stockholm

Forskare som genomför projektet
Anna-Karin Welmer

Projekttitel

Effekt av träning under akut sjukhusvistelse på funktionell förmåga för patienter 75 år och äldre–en pilotstudie

Uppgifter om ansökan
Ansökan om ändring inkom till Etikprövningsmyndigheten 2021-12-16 och blev valid 2021-12-21. Grundansökan med diarienummer 2020-06505 blev godkänd 2021-02-22 av Etikprövningsmyndigheten.

Ändringen avser tillägg av två studiecentra, breddning av inklusionskriterierna, att styrketräningen i interventionen kan göras med vikter, samt tillägg av mätning av vadomfång.

2021-06788-02-225388
2021-06788-02

Etikprövningsmyndigheten beslutar enligt nedan.

BESLUT

Etikprövningsmyndigheten godkänner den forskning som anges i ansökan.

På Etikprövningsmyndighetens vägnar

Håkan Löfgren
Ordförande

Beslutet har fattats av följande personer:

Ordförande
Håkan Löfgren

Beslutet har fattats efter föredragning av vetenskaplig sekreterare
Göran Holst

Beslutet sänds till

Ansvarig forskare: Anna-Karin Welmer

2021-06788-02-225388
2021-06788-02
