# Supplementary material for: Feasibility of the “Preventing functional decline in acutely hospitalized older patients (PREV_FUNC)” study—A three-armed randomized controlled pilot trial
Source: PLoS One. 2024 Jun 21;19(6):e0304570. doi: 10.1371/journal.pone.0304570 (PMC11192352; doi:10.1371/journal.pone.0304570)
Supplement: S4 File — (DOCX) [file pone.0304570.s005.docx]

Dnr 2021-06788-02

Lund department 1 medicine

**DECISION**

2022-01-13

**Research Principal**

Stockholm Region

**Researcher carrying out the project**

Anna-Karin Welmer

**Project title**

Effect of exercise during acute hospital stay on functional ability for patients 75 years and older - a pilot study

**Information about the application**

The application for change was submitted to the Ethics Review Authority 2021-12-16 and became valid 2021-12-21.

The basic application with registration number 2020–06505 was approved 2021-02-22 by

The Ethics Review Authority.

The change concerns the addition of two study centers, broadening of the inclusion criteria, that strength training in the intervention can be done with weights, as well as the addition of measurement of calf circumference.

The Ethics Review Authority decides as below.

**DECISION**

The Ethics Review Authority approves the research specified in the application.

­­­­­­­­

On behalf of the Ethics Review Authority

Håkan Löfgren

President

The decision has been made by the following people:

**President**

Håkan Löfgren

**The decision was made after a presentation by the scientific secretary**

Göran Holstr

**The decision is sent to**

Responsible researcher: Anna-Karin Welmer
