# Supplementary material for: Feasibility of the “Preventing functional decline in acutely hospitalized older patients (PREV_FUNC)” study—A three-armed randomized controlled pilot trial
Source: PLoS One. 2024 Jun 21;19(6):e0304570. doi: 10.1371/journal.pone.0304570 (PMC11192352; doi:10.1371/journal.pone.0304570)
Supplement: S5 File — (PDF) [file pone.0304570.s006.pdf]

Effekt av träning under akut sjukhusvistelse på funktionell förmåga för patienter 75 år och äldre–en pilotstudie

2021-06788-02
Ansökan om ändring
Ändringsansökan
Avvakta avgift - inkommen ansökan

Anna-Karin Welmer

1.1.1. Ange diarienummer och beslutsdatum på den tidigare godkända grundansökan.

2020-06505

2021-02-22

1.1.1.1. Ange vilken nämnd/myndighet som behandlade grundansökan

Etikprövningsmyndigheten (beslut fr.o.m. 2019)

1.1.2.1 Ska projektets titel bytas?

Nej

1.1.3. Ange diarienummer och beslutsdatum för eventuella tidigare ändringsansökningar och ge en kort summering av vad de avsåg. Om antalet tidigare ändringar överstiger fem ska en separat förteckning över ändringsansökningarna biläggas.

Dnr 2021-04736, Beslutsdatum 2021-11-02. Den tidigare ändringsansökan avsåg att tillägg av kvalitativa intervjuer för att utforska äldre personers uppfattning och erfarenhet av fysisk aktivitet och träning under sjukhusvistelsen. Vi planerar att intervjua runt 20–30 äldre personer och intervjuerna kommer att genomföras under sjukhusvistelsen. Datainsamlingen kommer att utföras av kvalificerad vårdpersonal, där forskningspersonerna kommer att intervjuas i 30–60 minuter vardera om sina uppfattningar och erfarenheter av fysisk aktivitet och inaktivitet under sjukhusvistelsen. Datainsamling sker med kvalitativa semistrukturerade djupintervjuer baserade på en intervjuguide med öppna frågor.

1.2.1 Ange ansvarig forskare för den tidigare godkända grundansökan.

Anna-Karin Welmer, e-post: anna-karin.welmer@ki.se

1.2.3. Ange forskningshuvudman för den tidigare godkända grundansökan.

Region Stockholm (232100-0032)

1.2.3.1 Behörig företrädare för grundansökans forskningshuvudman

Carina Metzner, verksamhetschef ME åldrande, Karolinska Universitetssjukhuset

1.2.3.2 Behörig företrädare grundansökan – titel som innebär ett verksamhetsansvar

Carina Metzner, verksamhetschef ME åldrande, Karolinska Universitetssjukhuset

Medverkande

Anne-Marie Boström

Linda Sandberg

1.3. Beskriv kortfattat den ändring av tidigare godkänd ansökan som planeras.

Ändringen avser tillägg av två studiecentra, Dalengeriatriken och Sabbatsbergsgeriatriken, Stockholm. Vidare avser ändringen att bredda inklusionskriterierna genom att ändra förmåga att gå till förmåga att resa sig upp från sittande, och genom att ta bort exklusionskriterierna demensdiagnos och en förväntad längd på sjukhusvistelsen mindre än tre dagar. Ändringen avser också att träningen i interventionsgrupp 2 (Individanpassad multikomponentträning) kan innehålla styrketräning med viktmanschetter och gummiband eller styrketräningsmaskiner (tidigare styrketräningsmaskiner). Ändringen avser också att lägga till mätning av vadomfång istället för bioimpedans (används för att definiera sarkopeni). Ändringarna visas med färgmarkeringar eller överstrykningar i forskningsplanen.

1.3.1. Sammanfattning av ändring

Ändringen avser tillägg av två studiecentra, breddning av inklusionskriterierna, att styrketräningen i interventionen kan göras med vikter, samt tillägg av mätning av vadomfång.

1.4. Ange de skäl som ligger till grund för den planerade ändringen.

Ändringen avser tillägg av två studiecentra, Dalengeriatriken och Sabbatsbergsgeriatriken, Stockholm. Detta görs dels för att undersöka effekten av interventionen i olika vårdmiljöer, vilket ökar den externa validiteten, dels för att få ett större underlag och öka inklusionstakten. Breddningen av inklusionskriterierna görs för att inkludera så många som möjligt från de patientgrupper som potentiellt kan ha nytta av interventionen. Ändringen avseende att träningen i interventionsgrupp kan innehålla styrketräning med viktmanschetter och gummiband eller styrketräningsmaskiner (tidigare styrketräningsmaskiner) gör för att underlätta genomförandet av studien. Likaså görs

tillägget att mäta vadomfång istället för att använda bioimpedans för att underlätta genomförandet av testerna.

1.5. Gör en värdering av hur förhållandet mellan riskerna och nyttan av projektet förändras med anledning av den planerade ändringen.

Samma risk/nyttoförhållande gäller för tillägget av nya studiecentra som för den ursprungliga ansökan. Tillägget kan öka den externa validiteten, vilket kan stärka studiens kvalitet. Datahantering och förvaring kommer att ske på samma sätt för de nya studiecentra som för det ursprungliga. Allt datamaterial är pseudonymiserat och inga personuppgifter kommer att kunna kopplas till forskningspersonerna. Resultat kommer enbart redovisas på gruppnivå. Breddningen av inklusionskriterierna gör att fler sköra forskningspersoner kan inkluderas. Risken för dessa personer skiljer sig inte från risken för de andra deltagarna. All träning och testning att utföras på ett säkert sätt där det vid behov finns en person att ta stöd av eller en stol eller brits att sätta sig på. Personalen som leder träningen och de som genomför testerna har god erfarenhet av att genomföra träning och testning av äldre på ett säkert sätt. Träning med viktmanschetter och gummiband utgör ingen ökad risk jämfört med träning i styrketräningsmaskiner. Mätning av vadomfång ingår i klinisk rutin för vissa av patienterna på klinikerna och utgör ingen risk.
Vi bedömer att riskerna med dessa tillägg är relativt små i förhållande till nyttan.

1.6. Kommer informationen till forskningspersonerna förändras med anledning av den planerade ändringen.

Ja

1.6.1. Beskriv hur informationen till forskningspersonerna förändras med anledning av den planerade ändringen.

Vi har lagt till att testerna inkluderar mätning av vadomfång. Vidare har vi tagit bort att testerna inkluderar mätning av kroppsammansättning och depressiva symptom. Mätning av depressiva symptom har tagits bort för att förkorta testningen. Testningen tar nu 20–30 minuter (tidigare 30–40). Detta har också ändrats.

1.7. Kommer annan information/bilagor förändras med anledning av den planerade ändringen.

Nej

1.8. Ange i förekommande fall vilka bilagor som bifogas ansökan.

Till ändringsansökan bifogas reviderad forskningspersonsinformation (bilaga 1) och reviderad forskningsplan (bilaga 2).

Bifoga relevanta bilagor

relevanta_bilagor-
ndringsanskan_Dnr_202006505._Bilaga_1_Informerat_samtycke_rev_december_2021.pdf
140.82KB

relevanta_bilagor-ndringsanskan_Dnr_202006505_Bilaga_2_december_2021.pdf
221.18KB

1.9 Huvudansvarig forskare för projektet (kontaktperson):

Anna-Karin Welmer

Signaturer

Signatur-huvudansvariga-forskare.pdf
28.1KB

Beslut och handlingar från Etikprövningsmyndigheten

Beslutsbrev och andra handlingar från Etikprövningsmyndigheten i relation till denna ansökan

2021-06788-02_Avgiftsavisering.pdf
34.33KB

Effekt av träning under akut sjukhusvistelse på funktionell förmåga för patienter 75 år och äldre–en pilotstudie

2021-06788-02-217211
2021-06788-02

Information om deltagande i forskningsprojekt

Vi vill fråga dig om du vill delta i ett forskningsprojekt. I det här dokumentet får du information om projektet och om vad det innebär att delta.

Vad är det för projekt och varför vill ni att jag ska delta?

Fysisk aktivitet är viktigt för en god hälsa. Enligt forskning kan fysisk träning under sjukhusvistelsen vara effektivt för att förbättra självständighet och funktion. Syftet är därför att utforska effekten av olika träningsinsatser riktade till äldre under sjukhusvistelse. För att delta ska du vara 75 år eller äldre samt uppfylla uppsatta kriterier för att delta i studien. Du tillfrågas om deltagande då du uppfyller kriterierna. Forskningshuvudman för projektet är Karolinska Universitetssjukhuset. Med forskningshuvudman menas den organisation som är ansvarig för studien.

Hur går studien till?

Ditt deltagande i denna forskningsstudie innebär att du i början och slutet av din vårdtid kommer att få genomföra tester och svara på enkäter. Testerna syftar till att undersöka gånghastighet, balans, benstyrka, greppstyrka, vadomfång, ~~kroppssammansättning, depressiva symptom,~~ kognitiva förmågor-samt förmåga till aktiviteter i det dagliga livet. Enkäterna syftar till att fråga dig hur du mår och hur du uppfattar din livskvalitet inom olika områden. Andra uppgifter som ålder, kön, din sociala situation, ditt medicinska tillstånd, vårdtiden och platsen dit du skrivs ut kommer att samlas in från din journal. Denna hälsoundersökning kommer att ta 20–30 minuter att genomföra.

Om du accepterar deltagande i studien så kommer du antingen få sedvanlig vård med sedvanlig rehabilitering tillsammans med fysioterapeut eller träning som innebär upp till 40 minuters träning upp till 4 gånger per dag. Övningar som kan ingå är styrke-, balans- och gångträning.

Möjliga följder och risker med att delta i studien

Test av balans kan utmana balansförmågan hos dig. För att inte riskera att du förlorar balansen och ramlar kommer det att finnas en fysioterapeut i nära anslutning till dig som kommer att vara beredd på att hjälpa dig.

Vad händer med mina uppgifter?

Uppgifter om din gånghastighet, balans, benstyrka, greppstyrka, vadomfång, ~~kroppssammansättning, depressiva symptom,~~ kognitiva förmågor, förmåga till aktiviteter i det dagliga livet, hur du mår och hur du uppfattar din livskvalitet inom olika områden, uppgifter om din ålder, kön, din sociala situation, ditt medicinska tillstånd, vårdtiden och platsen dit du skrivs ut till kommer skyddas av sekretess enligt offentlighets- och sekretesslagen, vilket innebär att inga obehöriga får ta del av uppgifterna. Det insamlade materialet kommer att kodas och data och kodnyckel kommer att förvaras separat inlåst enligt sedvanlig praxis. Det

betyder att ingen utom forskningsansvariga vid behov kan identifiera dig som person. Alla som arbetar med studien har tystnadsplikt. Samtliga resultat från studien kommer att presenteras på statistisk gruppnivå. Om vi vid hälsoundersökningen upptäcker något som behöver följas upp, blir du erbjuden kontakt med hälso- och sjukvårdspersonal för utredning.

Ansvarig för dina personuppgifter är Karolinska Universitetssjukhuset som forskningshuvudman. Enligt EU:s dataskyddsförordning har du rätt att kostnadsfritt få ta del av de uppgifter om dig som hanteras i studien, och vid behov få eventuella fel rättade. Du kan också begära att uppgifter om dig raderas samt att behandlingen av dina personuppgifter begränsas. Om du vill ta del av uppgifterna ska du kontakta Anna-Karin Welmer, Karolinska Institutet, Institutionen för fysioterapi, Alfred Nobels allé 23, 141 83 Huddinge, email: anna-karin.welmer@ki.se. Kodnyckeln kommer att förstöras efter 10 år. Efter det är det inte möjligt att lämna ut något registerutdrag. De uppgifter du lämnar skyddas enligt 24 kap. 8$ offentlighets- och sekretesslagen (2009:400) samt EU:s dataskyddsförordning, GDPR. Dataskyddsombud nås på dataskyddsombud.karolinska@sll.se. Om du är missnöjd med hur dina personuppgifter behandlas har du rätt att ge in klagomål till Datainspektionen, som är tillsynsmyndighet.

2021-06788-02-217211
2021-06788-02

Hur får jag information om resultatet av studien?

Studieresultaten kommer att redovisas på gruppnivå och publiceras i vetenskapliga tidskrifter, populärvetenskaplig skrift och på konferenser nationellt och internationellt. Din identitet kommer inte att kunna spåras i dessa redovisningar. Om du som forskningsperson vill få ut dina resultat går det bra att kontakta projektansvarig forskare. Om du inte vill ta del av dina resultat så kan du säga till innan studien startar.

Försäkring och ersättning

Ersättning utgår inte. Patientförsäkringen täcker ditt deltagande i studien.

Deltagandet är frivilligt

Ditt deltagande är frivilligt och du kan när som helst välja att avbryta deltagandet. Om du väljer att inte delta eller vill avbryta ditt deltagande behöver du inte uppge varför, och det kommer inte heller att påverka din framtida vård eller behandling.

Om du vill avbryta ditt deltagande ska du kontakta den ansvariga för studien (se nedan).

Ansvariga för studien

Om du önskar ytterligare information kontakta då någon av nedanstående:

Anna-Karin Welmer
Lektor, docent, fysioterapeut
Email: anna-karin.welmer@ki.se
08–524 888 04

Anne-Marie Boström
Lektor, docent, sjuksköterska
Email: anne-marie.bostrom@ki.se
0760–519513

Effekt av träning under akut sjukhusvistelse på funktionell förmåga för patienter 75 år och äldre–en pilotstudie

Samtycke till att delta i studien

Jag har fått muntlig och skriftlig informationen om studien och har haft möjlighet att ställa frågor. Jag får behålla den skriftliga informationen.

☐ Jag samtycker till att delta i studien Effekt av träning under akut sjukhusvistelse på funktionell förmåga för patienter 75 år och äldre–en pilotstudie

☐ Jag samtycker till att uppgifter om mig behandlas på det sätt som beskrivs i forskningspersonsinformationen.

Plats och datum	Underskrift

2021-06788-02-217211
2021-06788-02

2021-06788-02-217212
2021-06788-02

Effect of exercise on functional decline in patients older than 75 years during acute hospitalization—a pilot study of a three-armed randomized trial

BACKGROUND

Hospitalization because of acute medical illness is associated with several negative health consequences in older adults, such as loss of independence in activities of daily living (ADL) and increased risk of dementia (1, 2). These health consequences may occur even if the illness that caused the admission is successfully treated.

While intended to bring benefits, a hospital stay is often accompanied by low levels of physical activity, which has been suggested to play a major role in causing the negative health consequences associated with hospitalization (3). A recent meta-analysis suggested that in-hospital exercise interventions are effective for the improvement of functional independence; however, not all types of exercise interventions seemed to provide the same effects (4). Further research is needed to compare the effectiveness of different types of exercise interventions in acutely hospitalized older patients (4).

There is evidence indicating that multicomponent interventions including both mobility and strengthening exercises may be more effective than single-component interventions (4). In a recent randomized controlled trial (RCT), Martinez-Velilla, et al. demonstrated that a multicomponent exercise program, consisting of individualized supervised progressive resistance, balance, and walking training, provided significant health benefits over usual care in acutely hospitalized patients aged 75 years and older (5). Also, in another recent RCT Ortiz-Alonso J, et al. showed that a simple multicomponent intervention solely consisting of walking and rising from a chair decreased the risk of ADL dependence in acutely hospitalized patients older than 75 years (6).

So far, it is not known whether such a simple multicomponent exercise program could yield similar health benefits to a more comprehensive intervention, such as the one by Martinez-Velilla, et al. Furthermore, there is insufficient evidence concerning the effect of multicomponent exercise interventions on other clinically relevant outcomes than ADL such as falls and re-admission rates (4).

Prior to conducting a larger trial, it is important to examine the feasibility of the planned intervention in a pilot study. This includes assessing the process, such as recruitment and retention rate, exercise compliance, and acceptability as well as evaluating safety and scientific aspects of the intervention (7).

To be able to better adapt exercise programs according to the older adults´ needs and preferences, we must first achieve a better understanding of their perceptions and experiences of physical activity and exercise. Previous research has indicated that older adults who are frail and ill may have different perceptions of physical activity and exercise than community-living older adults in general (8). Åhlund, et al. (9) explored perceptions of physical activity and exercise among older adults who had been hospitalized because of acute medical illness. The interviews were however conducted a few months after discharge. To the best of our knowledge, no previous study has explored perceptions and experiences of physical activity and exercise among older adults during hospitalization.

OBJECTIVE. The aim of this pilot study is to evaluate the feasibility of a three-armed RCT, designed to evaluate if exercise interventions during acute hospitalization have effects over usual care concerning functional and health-related outcomes in patients aged 75 years and older, and if individualized and adapted prescription of multicomponent exercise to each patient is more effective than an intervention including simple physical exercise. To achieve a

deeper understanding and to better adapt and tailor the intervention, we also aim to explore the older adults' perceptions and experiences of physical activity and exercise.

Research questions:

1. How feasible is the RCT in terms of recruitment and retention rate, exercise compliance, acceptability, safety, and variance of the treatment effects?
2. How do patients aged 75 years and older perceive physical activity and exercise during acute hospitalization and what are their experiences of physical activity and exercise?

METHODS

Participants and study design. The study will be a tree-armed RCT, conducted in accordance with the Consolidated Standards of Reporting Trials (CONSORT) (10). Patients aged 75 years and older, admitted to a geriatric acute care ward at the Karolinska University Hospital, Dalengeriatriken, or Sabbatsbergsgeriatriken, Stockholm will be included if they are able to ~~walk (with or without personal support)~~ stand up from a sitting position and communicate and collaborate with the research team (e.g., patients with severe confusion will be excluded). We will exclude patients with ~~a diagnosis of dementia,~~ terminal illness or any major medical condition that contraindicates exercise~~, those with an expected length of hospitalization less than 3 days,~~ or those previously included in the study. We expect that 6-8 patients will be eligible for inclusion in the study per week. The pilot study will include around 24-30 participants per hospital (8-10 per group and hospital).

2021-06788-02-217212

The patients will be eligible to be included in the control group or one of the intervention groups in a time-dependent manner: patients will be recruited to the control group during one period, to intervention group 1 during another period, and to intervention group 2 during a third period. This design has been chosen to enable blinding of participants to group allocation. The order of the groups will be randomly selected for each ward/hospital. The staff that performs the examinations will also be blinded to group allocation. The participants will be invited to participate in the study as soon as possible after admission (within 24 hours during weekdays). The intervention will start the same day as the inclusion or the day after and continue until hospital discharge (including weekends).

This application only concerns the pilot study. A separate application for ethical approval will be submitted for the main RCT. To achieve a deeper understanding of how to be able to better tailor and adapt the intervention, we also aim to explore the older adults' perceptions and experiences of physical activity and exercise as part of the pilot phase of the study.

Intervention

Intervention group 1 (simple exercise program) (6)**.** The intervention will include up to four sessions per day (total duration 20-30 minutes/day): in the morning, before lunch, after lunch and in the evening. This intervention consists of chair stand exercises and walking along the corridor of the ward. The morning and evening sessions will be led by nurses, nursing assistants or physiotherapists and, when suitable, it will be performed in connection with the patient getting dressed or undressed. The day sessions will be led by physiotherapists, and when possible, it will be arranged as group training.

Intervention group 2 (individualized and adapted prescription of multicomponent exercise). This intervention consists of two daily sessions (morning and evening) of 20 minutes' duration each. The morning session includes individualized supervised progressive resistance, balance, and walking training exercises, tailored to each participant's capacity. The resistance

training includes using resistance training machines, weight cuffs, or fitness bands, involving mainly lower-extremity muscles (squats rising from a chair, leg press, and bilateral knee extension or similar exercises) and chest press, aiming at 2 to 3 sets of 8 to 10 repetitions on 30-60% of the 1-repetition maximum. Balance and gait exercises includes semi tandem foot standing, line walking, stepping practice, walking with small obstacles, proprioceptive exercises on unstable surfaces, altering the base of support and weight transfer. The morning session will be led by physiotherapists, and when possible, it will be arranged as group training. The evening session consists of functional exercises using light loads, such as knee extension and flexion, hip abduction, and daily walking along the corridor of the ward. The evening sessions will be led by nurses, nursing assistants or physiotherapists.

Control group. The control group will receive usual hospital care, which includes physical rehabilitation when needed.

Evaluation of feasibility. The feasibility will be examined in terms of recruitment and retention rate, exercise compliance, acceptability, safety, and variance of the treatment effects. Recruitment rate will be evaluated as the ratio between the number of eligible and included participants, retention rate is defined as the proportion of participants that complete the trial period, and exercise compliance as the ratio between the number of planned and attended exercise sessions. The time for each attended session will also be registered. Acceptability will be determined using a survey evaluating the participants' satisfaction with the exercise interventions. Treatment safety will be assessed by registering adverse events such as falls during the hospital stay. Exercise compliance and treatment safety will be recorded in a logbook for each participant.

2021-06788-02-217212
2021-06788-02

Variance of the treatment effects will be calculated based on the outcomes for the RCT. The primary outcomes are:

1. Change in mobility from admission to discharge, assessed by the Short Physical Performance Battery (SPPB) (11), which includes walking speed, balance and leg strength.
2. Change in ADL function from ~~two weeks prior to~~ admission ~~(assessed retrospectively)~~ to discharge, assessed by the Barthel Index of independence in ADL (12).

Secondary outcomes are change from admission to discharge in grip strength (assessed by the JAMAR hand dynamometer), health-related quality of life (assessed by the EuroQol–5 Dimension, EQ-5D) (12), and sarcopenia (defined by grip strength and calf circumference).

Data will also be collected at baseline on nutritional status (by the Mini Nutritional Assessment-Short Form, MNA-SF) (13), frailty (by the Clinical frailty scale) (14), and on length of hospital stay and discharge destination (proportion of participants discharged to their own home) at discharge ~~depressive symptoms (by the Montgomery-Åsberg Depression Rating Scale, MADRS) (13),~~ and cognition (by the Montreal Cognitive Assessment, MoCA) (14). Furthermore, we will collect data on age, sex, cohabitation status, reason for hospital admission, falls in the previous year, comorbidities, and prescribed medications from patient records. Type and amount of nutritional supplementation consumed during the hospitalization (if applicable) will be recorded in a logbook.

The results from all data will be depicted using descriptive statistics. The variance and differences in outcomes between the groups before and after the intervention will be evaluated with the Probabilistic Index (15). It reports the probability that the outcome of a randomly selected study participant in one group is higher than the outcome of another randomly selected study participant in another group. Furthermore, the results from the pilot study will be used to calculate power of the planned RCT (power calculations will be based on the

Barthel Index and the SPPB). Statistical analyses will be performed with Stata (StataCorp, TX, USA).

Qualitative data collection. The same inclusion criteria will be used to select participants for the interviews as for the pilot study, and the participants will be recruited from the same department. Participants for the interviews will however be recruited after the pilot study, i.e., those included in the intervention will not be selected for interviews. Purposive sampling will be used when selecting respondents for the interviews to ensure representation of a broad spectrum of participants from the target groups (e.g., based on age, sex, and frailty status). We expect to interview around 20-30 participants to reach saturation of the data. The interviews will be conducted during the hospital stay.

The interviews will be carried out during the autumn of 2021. The data collection will be performed by qualified health care staff, where the informants will be interviewed for 30-60 minutes each about their perceptions and experiences of physical activity and inactivity during the hospital stay. Data collection is done with qualitative semi-structured in-depth interviews based on an interview guide with open-ended questions. Data will be analyzed using qualitative content analysis (16).

RELEVANCE

Preservation of mobility and independence in ADL has been reported to be highly desired by older adults, even more than longevity (17). Older adults account for a disproportionately high percentage of hospital admissions and more than half of the hospital bed days in European countries (18). The number of hospital admissions and its associated health consequences such as ADL dependence and mobility limitation are likely to increase as the population age, leading to considerable costs for society as well as individual suffering. Identifying the most efficient interventions to reduce health consequences of acute hospitalization in older adults is thus highly relevant from clinical and public health perspectives. Evaluating the feasibility of the RCT prior to conducting a larger trial is an essential step to enhance the likelihood of success of the main study (7).

2021-06788-02-217212
2021-06788-02

TIME PLAN

Data for the interview study will be collected during autumn 2021 to spring 2022 and analyzed during spring 2022. Data for the pilot study will be collected and analyzed during spring 2022. The results will be presented in scientific papers and used to further plan the main RCT.

REFERENCES

1. Loyd C, Markland AD, Zhang Y, Fowler M, Harper S, Wright NC, et al. Prevalence of Hospital-Associated Disability in Older Adults: A Meta-analysis. Journal of the American Medical Directors Association 2020 Apr;21(4):455-+.
2. Ehlenbach WJ, Hough CL, Crane PK, Haneuse S, Carson SS, Curtis JR, et al. Association Between Acute Care and Critical Illness Hospitalization and Cognitive Function in Older Adults. Jama-Journal of the American Medical Association 2010 Feb;303(8):763-70.
3. Pavon JM, Sloane RJ, Pieper CF, Colon-Emeric CS, Cohen HJ, Gallagher D, et al. Accelerometer-Measured Hospital Physical Activity and Hospital-Acquired Disability in Older Adults. Journal of the American Geriatrics Society 2020 Feb;68(2):261-5.
4. Valenzuela PL, Morales JS, Castillo-Garcia A, Mayordomo-Cava J, Garcia-Hermoso A, Izquierdo M, et al. Effects of exercise interventions on the functional status of

acutely hospitalised older adults: A systematic review and meta-analysis. Ageing Research Reviews 2020 Aug;61.
5. Martinez-Velilla N, Casas-Herrero A, Zambom-Ferraresi F, de Asteasu MLS, Lucia A, Galbete A, et al. Effect of Exercise Intervention on Functional Decline in Very Elderly Patients During Acute Hospitalization A Randomized Clinical Trial. Jama Internal Medicine 2019 Jan;179(1):28-36.
6. Ortiz-Alonso J, Bustamante-Ara N, Valenzuela PL, Vidan-Astiz M, Rodriguez-Romo G, Mayordomo-Cava J, et al. Effect of a Simple Exercise Program on Hospitalization-Associated Disability in Older Patients: A Randomized Controlled Trial. Journal of the American Medical Directors Association 2020 Apr;21(4):531-+.
7. Thabane L, Ma J, Chu R, Cheng J, Ismaila A, Rios LP, et al. A tutorial on pilot studies: the what, why and how. Bmc Medical Research Methodology 2010 Jan;10.
8. Baert V, Gorus E, Mets T, Geerts C, Bautmans I. Motivators and barriers for physical activity in the oldest old: A systematic review. Ageing Research Reviews 2011 Sep;10(4):464-74.
9. Ahlund K, Oberg B, Ekerstad N, Back M. A balance between meaningfulness and risk of harm - frail elderly patients' perceptions of physical activity and exercise - an interview study. Bmc Geriatrics 2020 Nov;20(1).
10. Levack WMM, Engkasan JP, Heinemann AW, Negrini S. A Review of CONSORT Guidelines About Comparison Groups With a Focused Discussion on Implications for Rehabilitation Clinical Trials. American Journal of Physical Medicine & Rehabilitation 2020 Mar;99(3):191-7.
11. Guralnik JM, Simonsick EM, Ferrucci L, Glynn RJ, Berkman LF, Blazer DG, et al. A short physical performance battery assessing lower-extremity function - association with self-reported disability and prediction of mortality and nursing-home admission. Journals of Gerontology 1994 Mar;49(2):M85-M94.
12. vanBennekom CAM, Jelles F, Lankhorst GJ, Bouter LM. Responsiveness of the Rehabilitation Activities Profile and the Barthel Index. Journal of Clinical Epidemiology 1996 Jan;49(1):39-44.
13. Kaiser MJ, Bauer JM, Ramsch C, Uter W, Guigoz Y, Cederholm T, et al. Validation of the Mini Nutritional Assessment short-form (MNAA (R)-SF): A practical tool for identification of nutritional status. J Nutr Health Aging. 2009 Sep;13(9):782-8.
14. Boyd CM, Landefeld CS, Counsell SR, Palmer RM, Fortinsky RH, Kresevic D, et al. Recovery of Activities of Daily Living in Older Adults After Hospitalization for Acute Medical Illness. Journal of the American Geriatrics Society 2008 Dec;56(12):2171-9.
15. De Neve J, Thas O. A Regression Framework for Rank Tests Based on the Probabilistic Index Model. Journal of the American Statistical Association 2015;511:1276-83.
16. Graneheim UH, Lundman B. Qualitative content analysis in nursing research: concepts, procedures and measures to achieve trustworthiness. Nurse Education Today 2004 Feb;24(2):105-12.
17. Welmer AK, Morck A, Dahlin-Lvanoff S. Physical Activity in People Age 80 Years and Older as a Means of Counteracting Disability, Balanced in Relation to Frailty. J Aging Phys Act. 2012 Jul;20(3):317-31.
18. Union. OE. Health at a Glance: Europe 2016. OECD Publishinghing 2016.

2021-06788-02-217212
2021-06788-02

Signering av etikprövningsansökan

Ansökan om ändring

Forskningshuvudman: Region Stockholm

Projekttitel: Effekt av träning under akut sjukhusvistelse på funktionell förmåga för patienter 75 år och äldre–en pilotstudie

I och med att ansökan undertecknas intygar du som är ansvarig forskare följande:

- Att den information som lämnas i ansökan om etikprövning och samtliga medföljande bilagor är riktig och fullständig.
- Att verksamhetsansvariga i samtliga medverkande verksamheter är informerade om forskningsprojektets innehåll och utförande och att de har samtyckt till att delta i studien.
- Att du säkerställt att det i samtliga medverkande verksamheter finns resurser som garanterar forskningspersonernas säkerhet och integritet vid genomförandet av den forskning som beskrivs i ansökan.
- Att du tagit del av Etikprövningsmyndighetens information om hantering av personuppgifter på myndighetens webbplats.

Ansvarig forskare har signerat.

Signerat av Anna-Karin Welmer 2021-12-16 11:50:02

2021-06788-02-217216
2021-06788-02

Avgiftsavisering

Etikprövningsmyndigheten har tagit emot din ansökan med titel Effekt av träning under akut sjukhusvistelse på funktionell förmåga för patienter 75 år och äldre–en pilotstudie om ändring. Ansökan har diarienummer 2021-06788-02 vilket alltid ska anges i framtida kontakter i ärendet.

Avgiften för ansökan om ändring, som är 2000 kronor, ska omgående betalas in enligt nedan:

- Inbetalning sker till bankgironummer 406-1107
- Vid inbetalning ska OCR-nummer 2021067880231 anges som referens.
- Inga andra bokstäver eller siffror får anges i raden för referens.

Först när ärendet kompletterats enligt ovan kommer vi att påbörja handläggningen.

Etikprövningsmyndigheten
Telefon: 010 - 475 08 00
Webbplats: www.etikprovning.se

2021-06788-02-217215
2021-06788-02
