# Supplementary material for: Feasibility of the “Preventing functional decline in acutely hospitalized older patients (PREV_FUNC)” study—A three-armed randomized controlled pilot trial
Source: PLoS One. 2024 Jun 21;19(6):e0304570. doi: 10.1371/journal.pone.0304570 (PMC11192352; doi:10.1371/journal.pone.0304570)
Supplement: S6 File — (PDF) [file pone.0304570.s007.pdf]

Effect of exercise during acute hospitalization on functional capacity of patients 75 years and older – a pilot study

2021-06788-02
Application for change
Amendment application
Wait for fee - received application

Anna-Karin Welmer

1.1.1. Enter the registration number and decision date on the basic application that previously has been approved.

2020-06505

2021-02-22

1.1.1.1. State which board/authority processed the basic application.

The Ethics Review Authority (decision from 2019 onwards)

1.1.2.1 Should the project title be changed?

No

1.1.3. Enter registration number and decision date for any previous ones change requests and give a brief summary of what they intended. About the number previous changes exceed five shall a separate list of the amendment applications are attached.

Registration number 2021-04736, Decision date 2021-11-02. The previous application for amendment intended the addition of qualitative interviews to explore older people's perception and experience of physical activity and exercise during the hospital stay. We plan to interview around 20–30 older people and the interviews will be conducted during the hospital stay. The data collection will be carried out by qualified healthcare staff, where the research subjects will be interviewed for 30–60 minutes each about their perceptions and experiences of physical activity and inactivity during the hospital stay. Data collection takes place with qualitative semi-structured in-depth interviews based on an interview guide with open-ended questions.

1.2.1 Enter the responsible researcher for the previously approved basic application.

Anna-Karin Welmer, e-mail: anna-karin.welmer@ki.se

1.2.3. Enter the research principal for the previously approved basic application.

Region Stockholm (232100-0032)

1.2.3.1 Authorized representative of the basic application's research principal

Carina Metzner, operations manager ME aging, Karolinska University Hospital

1.2.3.2 Authorized representative basic application – title that implies a operational responsibility

Carina Metzner, operations manager ME aging, Karolinska University Hospital

Participating

Anne-Marie Boström
Linda Sandberg

1.3. Briefly describe the change to the previously approved application that is planned.

The change refers to the addition of two study centers, Dalengeriatriken and Sabbatsbergs-geriatriken, Stockholm. Furthermore, the change intends to broaden the inclusion criteria by changing the ability to walk to the ability to stand up from sitting, and by removing the exclusion criteria dementia diagnosis and an expected length of hospital stay of less than three days. The change also means that training in intervention group 2 (Individualized multi-component training) can include strength training with weight cuffs and rubber bands or strength training machines (formerly strength training machines). The change also intends to add measurement of calf circumference instead of bioimpedance (used to define sarcopenia). The changes are shown with color markings or strikeouts in the research plan.

1.3.1. Summary of change

The change refers to the addition of two study centers, broadening of the inclusion criteria, that strength training in the intervention can be done with weights, as well as the addition of measurement of calf circumference.

1.4. State the reasons underlying the planned change.

The change refers to the addition of two study centers, Dalengeriatriken and Sabbatsbergs-geriatriken, Stockholm. This is partly done to investigate the effect of the intervention in different care environments, which increases the external validity, partly to obtain a larger base and increase the rate of inclusion. The widening of the inclusion criteria are made to include as many as possible from the patient groups that could potentially benefit from the intervention. The change regarding the training in the intervention group may include strength training with weight cuffs and rubber bands or strength training machines (formerly strength training machines) do to facilitate the conduct of the study. The same is done

the addition of measuring calf circumference instead of using bioimpedance to facilitate implementation of the tests.

1.5. Make an assessment of how the relationship between the risks and benefits of the project changes due to the planned change.

The same risk/benefit ratio applies to the addition of new study centers as to the original basic application. The addition can increase the external validity, which can strengthen the quality of the study. Data management and storage will be done in the same way for the new study centers as for the original one. All data material is pseudonymised and no personal data will be linked the research subjects. Results will only be reported at group level. The widening of the inclusion criteria means that more frail research subjects can be included. The risk to these people does not differ from the risk of the other participants. All training and testing are carried out in a safely way where, if necessary, there is a person to support or a chair or bunk to sit on. The staff who lead the training and those who carry out the tests have good experience in carrying out training and testing elderly safely. Training with weight cuffs and rubber bands does not constitute an increased risk compared to training in strength training machines. Measurement of calf circumference is part of clinical routine for some of the patients in the clinics and does not pose a risk. We assess that the risks of these supplements are relatively small in relation to the benefit.

1.6. Will the information to the research subjects change due to the planned change.

Yes

1.6.1. Describe how the information to the research subjects changes with reason to the planned change.

We have added that the tests include measurement of calf circumference. Furthermore, we have removed those tests that includes measurement of body composition and depressive symptoms. Measurement of depressives symptoms have been removed to shorten testing. Testing now takes 20–30 minutes (previously 30–40). This has also been changed.

1.7. Will other information/appendices change because of the planned change.

No

1.8. If applicable, indicate which attachments are attached to the application.

Information to research persons (appendix 1) and the research plan (appendix 2) are revised are attached to the amendment application.

Attach relevant attachments

relevanta_bilagor-

ndringsanskan_Dnr_202006505._Bilaga_1_Informerat_samtycke_rev_december_2021.pdf

140.82KB

relevanta_bilagor-ndringsanskan_Dnr_202006505_Bilaga_2_december_2021.pdf

221.18KB

1.9 Principal researcher for the project (contact person):

Anna-Karin Welmer

Signatures

Signature-principal-responsible-researcher.pdf

28.1KB

Decisions and documents from the Ethics Review Authority

Decision letter and other documents from the Ethics Review Authority in relation to this application

2021-06788-02_Fee notification.pdf

34.33KB

Effect of exercise during acute hospitalization on functional capacity of patients 75 years and older–a pilot study

Information about participation in research project
We would like to ask you if you would like to participate in a research project. In this document you will receive information about the project and what it means to participate.

What kind of project is it and why do you want me to participate?
Physical activity is important for good health. According to research, physical exercise during hospitalization can be effective in improving independence and function. The aim is therefore to explore the effect of different exercise interventions during hospital stay. To participate, you must be 75 years of age or older and meet set criteria for participating in the study. You are asked to participate since you meet the criteria. The research principal for the project is Karolinska University Hospital. Research principal means the organization responsible for the study.

How is the study conducted?
Your participation in this research study will mean that at the beginning and end of your hospital stay, you will be asked to do tests and answer questionnaires. The tests aim to examine walking speed, balance, leg strength, grip strength, arm, and calf circumference, as well as ability to perform activities of daily living. The questionnaires aim to ask you how you perceive your health and quality of life in various areas. Other information such as age, sex, your medical condition, length of hospital stay, and place of discharge will be collected from your medical record. This examination will take approximately 20-30 minutes to complete.

If you choose to participate in the study, then you will either receive usual care with usual rehabilitation or training which means up to 40 minutes of training divided into a number of occasions per day (up to 4). Exercises that can be included are strength, balance, and walking.

Possible consequences and risks of participating in the study
Tests of balance can challenge your balance. In order not to risk losing your balance and fall, there will be a person close to you who will be ready to assist you.

What will happen to my data?
Information about your walking speed, balance, leg strength, grip strength, calf size, ability to perform activities of daily living, how you feel and how you perceive your quality of life in various areas, information about your age, gender, your social situation, your medical permission, the duration of care and the place to which you are discharged will be protected by confidentiality according to the Publicity and Secrecy Act, which means that no unauthorized persons may access the information. The collected material will be coded, and the data and code key will be kept separately locked according to standard practice. This

means that no one except the responsible researchers can identify you as a person if necessary. Everyone who works on the study is bound by confidentiality. All results from the study will be presented at group level. If during the examination we discover something that needs to be followed up, we can arrange contact with the relevant care facility.

Responsible for your personal data is Karolinska Institutet as the research principal. According to the EU's data protection regulation, you have the right to access the information about you, that is handled in the study, free of charge, and if necessary to have any errors corrected. You can also request that information about you be deleted and that the processing of your personal data be restricted. If you want to access the data, please contact Anna-Karin Welmer, Karolinska Institutet, Department of Physiotherapy, Alfred Nobels allé 23, 141 83 Huddinge, Sweden, telephone number: +46 8 524 888 04. The code key will be destroyed after 10 years. Thereafter, it is not possible to hand out any register extract. The information you provide is protected according to chapter 24. paragraph 8, of the Swedish Publicity and Privacy Act (2009:400) and the EU's data protection regulation, GDPR. The Data Protection Officer can be reached at dataskyddsombud.karolinska@sll.se. If you are dissatisfied with the way your personal data is processed, you have the right to file a complaint with the Swedish Authority for Privacy Protection (IMY), which is the supervisory authority.

How do I get information about the results of the study?
The study results will be reported at group level and published in scientific journals, popular science literature and at conferences nationally and internationally. Your identity will not be traceable in these reports. If you as a research participant want to get your results out, please contact the researcher responsible for the project. If you do not want to share your results, you can let us know before the study starts.

Insurance and compensation
Compensation is not eligible. Patient insurance covers your participation in the study.

Participation is voluntary
Your participation is voluntary, and you can choose to cancel your participation at any time. If you choose not to participate or wish to cancel your participation, you do not need to state why, and it will not affect your future care or treatment. If you wish to cancel your participation, please contact the responsible researchers (see below).

Responsible for the study
For further information, please contact:

Anna-Karin Welmer
Senior Lecturer, Associate Professor, Physiotherapist
anna-karin.welmer@ki.se
08–524 888 04

Anne-Marie Boström
Senior Lecturer, Associate Professor, RN
anne-marie.bostrom@ki.se
+46 760 519513

Effect of exercise during acute hospitalization on functional capacity of patients 75 years and older–a pilot study

Consent to participate in the study

I have received oral and written information about the study and have had the opportunity to ask questions. I get to keep the written information.

☐ I agree to participate in the study *Effect of exercise during acute hospitalization on functional capacity of patients 75 years and older–a pilot study*

☐ I agree to data about me being collected and processed in the manner described in the research participant information.

Place and date	Signature
	Name clarification

Effect of exercise on functional decline in patients older than 75 years during acute hospitalization—a pilot study of a three-armed randomized trial

BACKGROUND

Hospitalization because of acute medical illness is associated with several negative health consequences in older adults, such as loss of independence in activities of daily living (ADL) and increased risk of dementia (1, 2). These health consequences may occur even if the illness that caused the admission is successfully treated.

While intended to bring benefits, a hospital stay is often accompanied by low levels of physical activity, which has been suggested to play a major role in causing the negative health consequences associated with hospitalization (3). A recent meta-analysis suggested that in-hospital exercise interventions are effective for the improvement of functional independence; however, not all types of exercise interventions seemed to provide the same effects (4). Further research is needed to compare the effectiveness of different types of exercise interventions in acutely hospitalized older patients (4).

There is evidence indicating that multicomponent interventions including both mobility and strengthening exercises may be more effective than single-component interventions (4). In a recent randomized controlled trial (RCT), Martinez-Velilla, et al. demonstrated that a multicomponent exercise program, consisting of individualized supervised progressive resistance, balance, and walking training, provided significant health benefits over usual care in acutely hospitalized patients aged 75 years and older (5). Also, in another recent RCT Ortiz-Alonso J, et al. showed that a simple multicomponent intervention solely consisting of walking and rising from a chair decreased the risk of ADL dependence in acutely hospitalized patients older than 75 years (6).

So far, it is not known whether such a simple multicomponent exercise program could yield similar health benefits to a more comprehensive intervention, such as the one by Martinez-Velilla, et al. Furthermore, there is insufficient evidence concerning the effect of multicomponent exercise interventions on other clinically relevant outcomes than ADL such as falls and re-admission rates (4).

Prior to conducting a larger trial, it is important to examine the feasibility of the planned intervention in a pilot study. This includes assessing the process, such as recruitment and retention rate, exercise compliance, and acceptability as well as evaluating safety and scientific aspects of the intervention (7).

To be able to better adapt exercise programs according to the older adults´ needs and preferences, we must first achieve a better understanding of their perceptions and experiences of physical activity and exercise. Previous research has indicated that older adults who are frail and ill may have different perceptions of physical activity and exercise than community-living older adults in general (8). Åhlund, et al. (9) explored perceptions of physical activity and exercise among older adults who had been hospitalized because of acute medical illness. The interviews were however conducted a few months after discharge. To the best of our

knowledge, no previous study has explored perceptions and experiences of physical activity and exercise among older adults during hospitalization.

OBJECTIVE. The aim of this pilot study is to evaluate the feasibility of a three-armed RCT, designed to evaluate if exercise interventions during acute hospitalization have effects over usual care concerning functional and health-related outcomes in patients aged 75 years and older, and if individualized and adapted prescription of multicomponent exercise to each patient is more effective than an intervention including simple physical exercise. To achieve a deeper understanding and to better adapt and tailor the intervention, we also aim to explore the older adults' perceptions and experiences of physical activity and exercise.

Research questions:

1. How feasible is the RCT in terms of recruitment and retention rate, exercise compliance, acceptability, safety, and variance of the treatment effects?
2. How do patients aged 75 years and older perceive physical activity and exercise during acute hospitalization and what are their experiences of physical activity and exercise?

METHODS

Participants and study design. The study will be a tree-armed RCT, conducted in accordance with the Consolidated Standards of Reporting Trials (CONSORT) (10). Patients aged 75 years and older, admitted to a geriatric acute care ward at the Karolinska University Hospital, Dalengeriatriken, or Sabbatsbergsgeriatriken, Stockholm will be included if they are able to stand up from a sitting position and communicate and collaborate with the research team (e.g., patients with severe confusion will be excluded). We will exclude patients with terminal illness or any major medical condition that contraindicates exercise, or those previously included in the study. We expect that 6-8 patients will be eligible for inclusion in the study per week. The pilot study will include around 24-30 participants per hospital (8-10 per group and hospital).

The patients will be eligible to be included in the control group or one of the intervention groups in a time-dependent manner: patients will be recruited to the control group during one period, to intervention group 1 during another period, and to intervention group 2 during a third period. This design has been chosen to enable blinding of participants to group allocation. The order of the groups will be randomly selected for each ward/hospital. The staff that performs the examinations will also be blinded to group allocation. The participants will be invited to participate in the study as soon as possible after admission (within 24 hours during weekdays). The intervention will start the same day as the inclusion or the day after and continue until hospital discharge (including weekends).

This application only concerns the pilot study. A separate application for ethical approval will be submitted for the main RCT. To achieve a deeper understanding of how to be able to better tailor and adapt the intervention, we also aim to explore the older adults' perceptions and

experiences of physical activity and exercise as part of the pilot phase of the study.

Intervention

Intervention group 1 (simple exercise program) (6). The intervention will include up to four sessions per day (total duration 20-30 minutes/day): in the morning, before lunch, after lunch and in the evening. This intervention consists of chair stand exercises and walking along the corridor of the ward. The morning and evening sessions will be led by nurses, nursing assistants or physiotherapists and, when suitable, it will be performed in connection with the patient getting dressed or undressed. The day sessions will be led by physiotherapists, and when possible, it will be arranged as group training.

Intervention group 2 (individualized and adapted prescription of multicomponent exercise). This intervention consists of two daily sessions (morning and evening) of 20 minutes' duration each. The morning session includes individualized supervised progressive resistance, balance, and walking training exercises, tailored to each participant's capacity. The resistance training includes using resistance training machines, weight cuffs, or fitness bands, involving mainly lower-extremity muscles (squats rising from a chair, leg press, and bilateral knee extension or similar exercises) and chest press, aiming at 2 to 3 sets of 8 to 10 repetitions on 30-60% of the 1-repetition maximum. Balance and gait exercises includes semi tandem foot standing, line walking, stepping practice, walking with small obstacles, proprioceptive exercises on unstable surfaces, altering the base of support and weight transfer. The morning session will be led by physiotherapists, and when possible, it will be arranged as group training. The evening session consists of functional exercises using light loads, such as knee extension and flexion, hip abduction, and daily walking along the corridor of the ward. The evening sessions will be led by nurses, nursing assistants or physiotherapists.

Control group. The control group will receive usual hospital care, which includes physical rehabilitation when needed.

Evaluation of feasibility. The feasibility will be examined in terms of recruitment and retention rate, exercise compliance, acceptability, safety, and variance of the treatment effects. Recruitment rate will be evaluated as the ratio between the number of eligible and included participants, retention rate is defined as the proportion of participants that complete the trial period, and exercise compliance as the ratio between the number of planned and attended exercise sessions. The time for each attended session will also be registered. Acceptability will be determined using a survey evaluating the participants' satisfaction with the exercise interventions. Treatment safety will be assessed by registering adverse events such as falls during the hospital stay. Exercise compliance and treatment safety will be recorded in a logbook for each participant.

Variance of the treatment effects will be calculated based on the outcomes for the RCT. The primary outcomes are:

1. Change in mobility from admission to discharge, assessed by the Short Physical Performance Battery (SPPB) (11), which includes walking speed, balance and leg strength.
2. Change in ADL function from admission to discharge, assessed by the Barthel Index of independence in ADL (12).

Secondary outcomes are change from admission to discharge in grip strength (assessed by the JAMAR hand dynamometer), health-related quality of life (assessed by the EuroQol–5 Dimension, EQ-5D) (12), and sarcopenia (defined by grip strength and calf circumference).

Data will also be collected at baseline on nutritional status (by the Mini Nutritional Assessment-Short Form, MNA-SF) (13), frailty (by the Clinical frailty scale) (14), and on length of hospital stay and discharge destination (proportion of participants discharged to their own home) at discharge, and cognition (by the Montreal Cognitive Assessment, MoCA) (14). Furthermore, we will collect data on age, sex, cohabitation status, reason for hospital admission, falls in the previous year, comorbidities, and prescribed medications from patient records. Type and amount of nutritional supplementation consumed during the hospitalization (if applicable) will be recorded in a logbook.

The results from all data will be depicted using descriptive statistics. The variance and differences in outcomes between the groups before and after the intervention will be evaluated with the Probabilistic Index (15). It reports the probability that the outcome of a randomly selected study participant in one group is higher than the outcome of another randomly selected study participant in another group. Furthermore, the results from the pilot study will be used to calculate power of the planned RCT (power calculations will be based on the Barthel Index and the SPPB). Statistical analyses will be performed with Stata (StataCorp, TX, USA).

Qualitative data collection. The same inclusion criteria will be used to select participants for the interviews as for the pilot study, and the participants will be recruited from the same department. Participants for the interviews will however be recruited after the pilot study, i.e., those included in the intervention will not be selected for interviews. Purposive sampling will be used when selecting respondents for the interviews to ensure representation of a broad spectrum of participants from the target groups (e.g., based on age, sex, and frailty status). We expect to interview around 20-30 participants to reach saturation of the data.

The interviews will be conducted during the hospital stay. The interviews will be carried out during the autumn of 2021. The data collection will be performed by qualified health care staff, where the informants will be interviewed for 30-60 minutes each about their perceptions and experiences of physical activity and inactivity during the hospital stay. Data collection is done with qualitative semi-structured in-depth interviews based on an interview guide with open-ended questions. Data will be analyzed using qualitative content analysis (16).

RELEVANCE

Preservation of mobility and independence in ADL has been reported to be highly desired by older adults, even more than longevity (17). Older adults account for a disproportionately high percentage of hospital admissions and more than half of the hospital bed days in European countries (18). The number of hospital admissions and its associated health consequences such as ADL dependence and mobility limitation are likely to increase as the population age, leading to considerable costs for society as well as individual suffering. Identifying the most efficient interventions to reduce health consequences of acute hospitalization in older adults is thus highly relevant from clinical and public health perspectives. Evaluating the feasibility of

the RCT prior to conducting a larger trial is an essential step to enhance the likelihood of success of the main study (7).

TIME PLAN

Data for the interview study will be collected during autumn 2021 to spring 2022 and analyzed during spring 2022. Data for the pilot study will be collected and analyzed during spring 2022. The results will be presented in scientific papers and used to further plan the main RCT.

REFERENCES

1. Loyd C, Markland AD, Zhang Y, Fowler M, Harper S, Wright NC, et al. Prevalence of Hospital-Associated Disability in Older Adults: A Meta-analysis. Journal of the American Medical Directors Association 2020 Apr;21(4):455-+.

2. Ehlenbach WJ, Hough CL, Crane PK, Haneuse S, Carson SS, Curtis JR, et al. Association Between Acute Care and Critical Illness Hospitalization and Cognitive Function in Older Adults. Jama-Journal of the American Medical Association 2010 Feb;303(8):763-70.

3. Pavon JM, Sloane RJ, Pieper CF, Colon-Emeric CS, Cohen HJ, Gallagher D, et al. Accelerometer-Measured Hospital Physical Activity and Hospital-Acquired Disability in Older Adults. Journal of the American Geriatrics Society 2020 Feb;68(2):261-5.

4. Valenzuela PL, Morales JS, Castillo-Garcia A, Mayordomo-Cava J, Garcia-Hermoso A, Izquierdo M, et al. Effects of exercise interventions on the functional status of acutely hospitalised older adults: A systematic review and meta-analysis. Ageing Research Reviews 2020 Aug;61.

5. Martinez-Velilla N, Casas-Herrero A, Zambom-Ferraresi F, de Asteasu MLS, Lucia A, Galbete A, et al. Effect of Exercise Intervention on Functional Decline in Very Elderly Patients During Acute Hospitalization A Randomized Clinical Trial. Jama Internal Medicine 2019 Jan;179(1):28-36.

6. Ortiz-Alonso J, Bustamante-Ara N, Valenzuela PL, Vidan-Astiz M, RodriguezRomo G, Mayordomo-Cava J, et al. Effect of a Simple Exercise Program on HospitalizationAssociated Disability in Older Patients: A Randomized Controlled Trial. Journal of the American Medical Directors Association 2020 Apr;21(4):531-+.

7. Thabane L, Ma J, Chu R, Cheng J, Ismaila A, Rios LP, et al. A tutorial on pilot studies: the what, why and how. Bmc Medical Research Methodology 2010 Jan;10.

8. Baert V, Gorus E, Mets T, Geerts C, Bautmans I. Motivators and barriers for physical activity in the oldest old: A systematic review. Ageing Research Reviews 2011 Sep;10(4):464-74.

9. Ahlund K, Oberg B, Ekerstad N, Back M. A balance between meaningfulness and risk of harm - frail elderly patients' perceptions of physical activity and exercise - an interview study. Bmc Geriatrics 2020 Nov;20(1).

10. Levack WMM, Engkasan JP, Heinemann AW, Negrini S. A Review of CONSORT Guidelines About Comparison Groups With a Focused Discussion on Implications for Rehabilitation Clinical Trials. American Journal of Physical Medicine & Rehabilitation 2020 Mar;99(3):191-7.

11. Guralnik JM, Simonsick EM, Ferrucci L, Glynn RJ, Berkman LF, Blazer DG, et al. A short physical performance battery assessing lower-extremity function - association with self-reported disability and prediction of mortality and nursing-home admission. Journals of Gerontology 1994 Mar;49(2):M85-M94.

12. vanBennekom CAM, Jelles F, Lankhorst GJ, Bouter LM. Responsiveness of the Rehabilitation Activities Profile and the Barthel Index. Journal of Clinical Epidemiology 1996 Jan;49(1):39-44.

13. Kaiser MJ, Bauer JM, Ramsch C, Uter W, Guigoz Y, Cederholm T, et al. Validation of the Mini Nutritional Assessment short-form (MNAA (R)-SF): A practical tool for identification of nutritional status. J Nutr Health Aging. 2009 Sep;13(9):782-8.

14. Boyd CM, Landefeld CS, Counsell SR, Palmer RM, Fortinsky RH, Kresevic D, et al. Recovery of Activities of Daily Living in Older Adults After Hospitalization for Acute Medical Illness. Journal of the American Geriatrics Society 2008 Dec;56(12):2171-9.

15. De Neve J, Thas O. A Regression Framework for Rank Tests Based on the Probabilistic Index Model. Journal of the American Statistical Association 2015;511:1276-83.

16. Graneheim UH, Lundman B. Qualitative content analysis in nursing research: concepts, procedures and measures to achieve trustworthiness. Nurse Education Today 2004 Feb;24(2):105-12.

17. Welmer AK, Morck A, Dahlin-Lvanoff S. Physical Activity in People Age 80 Years and Older as a Means of Counteracting Disability, Balanced in Relation to Frailty. J Aging Phys Act. 2012 Jul;20(3):317-31.

18. Union. OE. Health at a Glance: Europe 2016. OECD Publishinghing 2016.

Signing of the ethics review application

Application for change

Research principal: Region Stockholm

Project title: Effect of exercise on functional decline in patients older than 75 years during acute hospitalization—a pilot study

By signing the application, you as the responsible researcher certify the following:

- That the information provided in the application for ethics review and all accompanying attachments are correct and complete.
- That operational managers in all participating operations are informed about the content and execution of the research project and that they have agreed to participate in the study.
- That you have ensured that in all participating businesses there are resources that guarantees the safety and integrity of the research subjects during its implementation research described in the application.
- That you have read the Ethics Review Authority's information on handling of personal data on the authority's website.

Responsible researcher has signed.

Signed by Anna-Karin Welmer 2021-12-16 11:50:02

Fee notification

The Ethics Review Authority has received your application with the title Effect of exercise on functional decline in patients older than 75 years during acute hospitalization—a pilot study of change. The application has registration number 2021-06788-02, which must always be stated in future contacts in the matter.

The fee for the application for change, which is SEK 2,000, must be paid immediately as below:

- Payment is made to bank giro number 406-1107
- When paying, OCR number 2021067880231 must be entered as a reference.
- No other letters or numbers may be entered in the line for reference.

Only when the matter has been completed as above will we begin processing.

The Ethics Review Authority

Telephone: 010 - 475 08 00

Website: www.etikprovning.se
