# Supplementary material for: Feasibility of the “Preventing functional decline in acutely hospitalized older patients (PREV_FUNC)” study—A three-armed randomized controlled pilot trial
Source: PLoS One. 2024 Jun 21;19(6):e0304570. doi: 10.1371/journal.pone.0304570 (PMC11192352; doi:10.1371/journal.pone.0304570)
Supplement: Attachment — Submitted filename: Response to Reviewers.docx [file pone.0304570.s008.docx]

Dear Academic Editor Sascha Köpke

Thank you for considering our manuscript "**Feasibility of the “Preventing functional decline in acutely hospitalized older patients (PREV_FUNC)” study – A three-armed randomized controlled pilot trial**" for publication in PLOS ONE. We have revised the manuscript based on the corrections suggested by the Academic Editor and the Reviewers. The respond to each comment can be found below. Our revisions in the manuscript can be followed by the “tracked changes” function.

Sincerely,
Linda Sandberg, PhD

Karolinska Institutet

Department of Neurobiology, Care Sciences and Society

Division of Physiotherapy, 23100

141 83 Huddinge, Sweden

Email: [linda.m.sandberg@ki.se](mailto:linda.m.sandberg@ki.se)

**Authors’ responses to Editor’s and Reviewers’ comments**

## **Comments from Journal requirements / Academic Editor**

**Comment**
1. When submitting your revision, we need you to address these additional requirements.

Please ensure that your manuscript meets PLOS ONE's style requirements, including those for file naming. The PLOS ONE style templates can be found at 
<https://journals.plos.org/plosone/s/file?id=wjVg/PLOSOne_formatting_sample_main_body.pdf> and 
<https://journals.plos.org/plosone/s/file?id=ba62/PLOSOne_formatting_sample_title_authors_affiliations.pdf>.

**Response**

We have carefully reviewed the requirements, corrected the names of the files (page 30), and made minor adjustments to the font used in the title (page 1, line 1-3).

**Comment**
2. We note that you have indicated that there are restrictions to data sharing for this study. PLOS only allows data to be available upon request if there are legal or ethical restrictions on sharing data publicly. For more information on unacceptable data access restrictions, please see <http://journals.plos.org/plosone/s/data-availability#loc-unacceptable-data-access-restrictions>.

**Response**

We appreciate the opportunity to respond to the prompts regarding data availability for our study. Please see our response below.

**Comment**
Before we proceed with your manuscript, please address the following prompts:

a) If there are ethical or legal restrictions on sharing a de-identified data set, please explain them in detail (e.g., data contain potentially identifying or sensitive patient information, data are owned by a third-party organization, etc.) and who has imposed them (e.g., a Research Ethics Committee or Institutional Review Board, etc.). Please also provide contact information for a data access committee, ethics committee, or other institutional body to which data requests may be sent.

**Response**

Ethical and Legal Restrictions: We acknowledge the importance of data sharing for scientific transparency and reproducibility. However, we must highlight that our dataset contains coded data from 39 participants. There is a key code that can be used to trace back the data to living individuals. According to the Swedish Archive Law, we need to keep the key code for at least 10 years post publication. The existence of the code means that the data are “pseudonymized personal data” as per European General Data Protection Regulation (GDPR) and cannot be published/shared openly. There is no other way to de-identify the data, given the small sample size, there always remains a risk of potential re-identification of individual participants. The European GDPR law on data protection and privacy imposes restrictions on openly sharing personal data. Our study has also been conducted in compliance with ethical guidelines, and the participants have not given their consent for their data to be made fully public. Sharing the data openly would not only constitute a GDPR breach but also a breach of the Swedish Ethical Approval law that has approved the research on the terms that personal data are protected as per GDPR and as per the conditions that the participants have agreed to.

For inquiries regarding access to the data, we would like to provide contact information for our Research Data Office at Karolinska Institutet: [rdo@ki.se](mailto:rdo@ki.se). They will handle requests for data access and ensure compliance with legal and ethical standards.

**Comment**
b) If there are no restrictions, please upload the minimal anonymized data set necessary to replicate your study findings to a stable, public repository and provide us with the relevant URLs, DOIs, or accession numbers. For a list of recommended repositories, please see
<https://journals.plos.org/plosone/s/recommended-repositories>. You also have the option of uploading the data as Supporting Information files, but we would recommend depositing data directly to a data repository if possible.

We will update your Data Availability statement on your behalf to reflect the information you provide.

**Response**

Data Availability Statement: In light of the restrictions outlined above, we propose updating the Data Availability Statement as follows:

"The data in the study are pseudonymized (coded) personal data, and European General Data Protection Regulation prohibits us from sharing this completely open. The dataset only includes data from 39 human research participants. Due to the small sample size, there is a risk of identification of individual participants even though the data is de-identified. Data is available upon request, and requests for access to the data can be put to our Research Data Office ([rdo@ki.se](mailto:rdo@ki.se)) at Karolinska Institutet."

We trust that this clarification addresses the concerns raised by the journal regarding data sharing. We remain committed to promoting transparency and scientific integrity while ensuring compliance with legal and ethical standards.

**Comment**
3. In the online submission form, you indicated that [Data Availability Statement: The data in the study are pseudonymised (coded) personal data, and Swedish legislation prohibits us from sharing this completely open. The dataset only includes data from 39 human research participants. Due to the small sample size risk of identification of individual participants exists if the data is shared, even though data is de-identified. Data is available upon request, and requests for access to the data can be put to our Research Data Office ([rdo@ki.se](mailto:rdo@ki.se)) at Karolinska Institutet.]. 
All PLOS journals now require all data underlying the findings described in their manuscript to be freely available to other researchers, either 1. In a public repository, 2. Within the manuscript itself, or 3. Uploaded as supplementary information.
This policy applies to all data except where public deposition would breach compliance with the protocol approved by your research ethics board. If your data cannot be made publicly available for ethical or legal reasons (e.g., public availability would compromise patient privacy), please explain your reasons on resubmission and your exemption request will be escalated for approval.

**Response**

Please see our response to point number 2a and 2b above.

**Comment**
4. Please include your full ethics statement in the ‘Methods’ section of your manuscript file. In your statement, please include the full name of the IRB or ethics committee who approved or waived your study, as well as whether or not you obtained informed written or verbal consent. If consent was waived for your study, please include this information in your statement as well.

**Response**

Thank you for addressing this matter. We have now incorporated an Ethics statement in which we have relocated the relevant text from the sections Study design and participants and Procedure (page 7, line 135-141). The statement now reads as follows “Ethical approval was obtained from *The Swedish Ethical Review Authority* Dnr. 2020-06505, and 2021-06788-02. All participants received verbal and written information and informed written consent was obtained before inclusion. The collected data were pseudonymized and stored in accordance with the General Data Protection Regulation. Only the responsible researchers had full access to the data.”

**Comment**
5. Please include captions for your Supporting Information files at the end of your manuscript, and update any in-text citations to match accordingly. Please see our Supporting Information guidelines for more information: <http://journals.plos.org/plosone/s/supporting-information>.

**Response**

We have corrected the names of the following files

From: **S1 File. English translation – Ethical approval 1.**
To: **S2 File. Ethical approval 1 – English translation.**

From: **S2 File. English translation – Ethical approval 2.**

To: **S4 File. Ethical approval 2 – English translation.**

We will add the following Supporting information files to the list in the manuscript and corrected the names of the files:

From: File Ethical approval 1.pdf

To: **S1 File. Ethical approval 1 – In Swedish.**

From: File Ethical approval 2.pdf

To: **S3 File. Ethical approval 2 – In Swedish.**

From: 2021-06788-02.pdf (Etikansökan på svenska 2021-06788-02)

To: **S5 File. Ethical application – In Swedish.**

From: File English transl 2021-06788-02.pdf )

To: **S6 File. Ethical application – English translation.**

**Comment**
6. Please review your reference list to ensure that it is complete and correct. If you have cited papers that have been retracted, please include the rationale for doing so in the manuscript text, or remove these references and replace them with relevant current references. Any changes to the reference list should be mentioned in the rebuttal letter that accompanies your revised manuscript. If you need to cite a retracted article, indicate the article’s retracted status in the References list and also include a citation and full reference for the retraction notice.

**Response**

Corrections have been made in the reference list. Reference 3 has been changed.

**Reviewers' comments
Comments**

1. Is the manuscript technically sound, and do the data support the conclusions?

The manuscript must describe a technically sound piece of scientific research with data that supports the conclusions. Experiments must have been conducted rigorously, with appropriate controls, replication, and sample sizes. The conclusions must be drawn appropriately based on the data presented.

Reviewer #1: Yes

Reviewer #2: Yes

2. Has the statistical analysis been performed appropriately and rigorously?

Reviewer #1: Yes

Reviewer #2: Yes

3. Have the authors made all data underlying the findings in their manuscript fully available?

The [PLOS Data policy](https://eur01.safelinks.protection.outlook.com/?url=http%3A%2F%2Fwww.plosone.org%2Fstatic%2Fpolicies.action%23sharing&data=05%7C02%7Clinda.m.sandberg%40ki.se%7C50018a3dc09142bc4b9608dc3c3a56f4%7Cbff7eef1cf4b4f32be3da1dda043c05d%7C0%7C0%7C638451468637272642%7CUnknown%7CTWFpbGZsb3d8eyJWIjoiMC4wLjAwMDAiLCJQIjoiV2luMzIiLCJBTiI6Ik1haWwiLCJXVCI6Mn0%3D%7C0%7C%7C%7C&sdata=ruTf2Cl4KO8%2F4F0D6%2FvCLKVlwkcS6nD3ID5XTfeDkB0%3D&reserved=0) requires authors to make all data underlying the findings described in their manuscript fully available without restriction, with rare exception (please refer to the Data Availability Statement in the manuscript PDF file). The data should be provided as part of the manuscript or its supporting information, or deposited to a public repository. For example, in addition to summary statistics, the data points behind means, medians and variance measures should be available. If there are restrictions on publicly sharing data—e.g. participant privacy or use of data from a third party—those must be specified.

Reviewer #1: Yes

Reviewer #2: No

4. Is the manuscript presented in an intelligible fashion and written in standard English?

PLOS ONE does not copyedit accepted manuscripts, so the language in submitted articles must be clear, correct, and unambiguous. Any typographical or grammatical errors should be corrected at revision, so please note any specific errors here.

Reviewer #1: Yes

Reviewer #2: Yes

**Response**: Thank you for taking the time to review the manuscript. Regarding question number 3, data availability, please see our response to Editor’s comments point number 2a and 2b above.

**Reviewer #1**

**Comment**

Feasibility of the “Preventing functional decline in acutely hospitalized older patients” study – A three-armed randomized controlled trial
This manuscript described a randomized controlled trial assessing recruitment and retention rates, intervention compliance, acceptability, and scientific feasibility of 3 different exercise programs for acutely ill older inpatients in geriatric units or clinics of 3 different hospitals in Sweden. The authors highlight that few studies have compared different exercise programs in the acute care setting among older patients.
I think overall this is a well-designed assessment and the authors did a very good job of writing up their findings.

**Response**

Thank you for reviewing our manuscript, and for your positive comments regarding the manuscript.

**Comment**

One question I have relates to inpatients with mild cognitive impairment (MCI). Other studies have shown that at least 1/3 of older inpatients have some level of cognitive impairment, and that patients with MCI are very limited in terms of physical function and mobility. Have the authors considered the impact of their interventions for patients with MCI? It seems these interventions are helpful for many but not all patients. Also, what happens when a patient develops delirium during hospitalization? How will the authors address this issue in the later RCT? The authors should explain why they are not assessing cognition at baseline using a standardized validated tool such as the Mini-cog. This is important not only for confirming the patient can provide written consent, but also for confirming cognition status. I think this piece of data should be prioritized during recruitment and enrollment for the later RCT and can be used to better understand the results of the study. If it is possible to include some patients with MCI, that would ultimately provide more evidence to present related to this trial. Alternatively, if the exercise programs are not appropriate for patients with MCI that should be clearly articulated and explained.

**Response**

Thank you for allowing us to address this important issue. Our inclusion criteria did not exclude participants with cognitive impairment; rather, they emphasized the participants’ ability to communicate and collaborate with the research staff. As stated in the Procedure section (page 7, line 147-150), “Consent was obtained by research staff with sensitivity, meaning that no one was enrolled in the study if they appeared to have difficulty understanding what was meant by informed consent or expressed discomfort regarding participating.”

However, we concur with the Reviewer that the absence of an objective measure of cognitive function represents a limitation in our study. To address this concern, we have included the following sentences in the discussion (page 21, line 429-431). “Our study did not incorporate a measure of cognitive function. Consequently, we cannot definitively conclude whether the intervention is feasible for older adults with cognitive impairment.”

**Comment**
The first reference was cited twice – reference 1 and reference 3 in the bibliography.

**Response**

Thank you for noticing this. This has been corrected (page 4, line 62 and 64, and page 25).

## **Reviewer #2**

**Comment**
This paper reports preliminary results from a feasibility study of in-hospital exercise. The manuscript is clearly written with intervention and analytical details well-documented. The major limitation is the small sample size. However, the authors clarified that the purpose of the study is just to examine the feasibility rather than intervention effectiveness. I only have a few minor suggestions:

**Response**

Thank you for your careful revision of our manuscript, and for helping us to further improve it. Please, see our responses to your comments below.

**Comment**
1. The study excludes individuals living in nursing homes. It would be helpful if the authors could provide an explanation for this exclusion, given that the intervention is conducted in hospitals.

**Response**

Thank you for allowing us to clarify this issue. To address this concern, we have included the following sentences in the discussion (page 22, line 431-436). “Furthermore, we excluded individuals living in nursing homes for two specific reasons. Firstly, a comparable exercise program to the simple exercise program had already been tested in nursing homes as part of a previous study [32]. Secondly, considering that participants in the Simple exercise program will be encouraged to continue their exercises at home during the full-scale trial, it was deemed more appropriate to include only those living in their own homes.”

32	Grönstedt, H., Vikström, S., Cederholm, T., Franzén, E., Luiking, Y. C., Seiger, Å., ... & Boström, A. M. (2020). Effect of sit-to-stand exercises combined with protein-rich oral supplementation in older persons: the older person's exercise and nutrition study. *Journal of the American Medical Directors Association*, *21*(9), 1229-1237.

**Comment**
2. In several instances, the purpose of the study is stated as examining the trends and variance of the outcome measures Does “variance” here actually mean “change” as no variance was reported? If so, please consider revise as the readers may confuse it with the statistical term variance.

**Response**: We acknowledge that the previous wording was unclear. In response, we have replaced the term “variance” with “95% confidence interval (CI)” throughout the manuscript. Additionally, we have included the following sentence under the data analysis section, on page 11, line 245-246: “The CI was utilized to describe the uncertainty associated with the point estimates for trend.”

**Comment**

3. Considering the small sample size, univariate quantile regression may not have sufficient power. If the intention is to compare the mean or median of the SPPB, I recommend considering non-parametric tests such as the Kruskal-Wallis test.

**Response**: Thank you for allowing us to clarify this issue. Quantile regression analysis was used to calculate the 95% CIs, not p-values. We have added the following sentences to the discussion, page 22, line 447-451: “Given the small sample size and the aim of feasibility studies, which is to assess the feasibility of a study rather than the effect of interventions, we made the decision not to present p-values for group differences. Instead, we utilized 95% CIs to describe the uncertainty associated with the point estimates for trend.”

**Comment**

4. When analyzing the SPPB and Barthel Index, will the sum scores be used? If so, please note that these scores are continuous rather than ordinal. Additionally, ordinal scales are technically a type of categorical variable. I suggest the authors review and clarify the definitions when describing the variables used for analysis.

**Response**

Thank you for pointing this out. We revised the text under Data analysis, page 11, line 242-246, as follows “The SPPB and Barthel Index were employed as continuous scales in univariate quantile regression models to assess median change by group in each outcome measure with 95% CI.”

**Comment**

5. The paper mentions that three participants were lost before the baseline assessment. It would be helpful to provide an explanation for the reasons behind this loss.

**Response**

Unfortunately, we have no documented information about these three participants who were lost before the baseline assessment.

**Comment**

6. Although the sample size for each group is small, it is still worth discussing whether the differences in sample characteristics and outcome scores at baseline and after the intervention are statistically significant based on non-parametric tests.

**Response**

We used quantile regression analysis, which is a semi-parametric test suitable for small and non-normally distributed samples. Due to the small sample size and the feasibility nature of the study, we chose to show 95% CI instead of p-values. To address this concern, we have included the following sentence in the discussion (page 23, line 463-464). “However, due to the limited sample size and the wide CIs, which indicate substantial uncertainty around the point estimates, the results need to be interpreted with caution.”
